# Serotonin 5-HT_2A_ and 5-HT_2C_ receptors differentially modulate the acquisition and expression of voluntary alcohol drinking in male mice

**DOI:** 10.3389/fnins.2025.1639344

**Published:** 2025-08-25

**Authors:** Sandy C. Simões, Livia N. Amorim, Yasmim A. Serra, Camila S. Abreu, Maria Clara E. Santana, João P. C. Leite, Carla M. Kaneto, Roberta C. A. Costa, Kaio R. Ferreira, Isabelle C. S. de Oliveira, Alexandre J. Oliveira-Lima, Agnieszka Sulima, Kenner C. Rice, Eduardo A. V. Marinho, Lais F. Berro

**Affiliations:** ^1^Department of Health Sciences, Universidade Estadual de Santa Cruz, Ilhéus, Brazil; ^2^Drug Design and Synthesis Section, Molecular Targets and Medications Discovery Branch, National Institute on Drug Abuse and the National Institute on Alcohol Abuse and Alcoholism, Bethesda, MD, United States; ^3^Department of Psychiatry and Human Behavior, Center for Innovation and Discovery in Addictions, University of Mississippi Medical Center, Jackson, MS, United States

**Keywords:** alcohol, serotonin, two-bottle choice, mice, 5-HT_2A_, 5-HT_2C_

## Abstract

**Introduction:**

Studies suggest that serotonin (5-HT) plays an important role in alcohol use disorder (AUD). While several receptor subtypes modulate the role of 5-HT in AUD, evidence suggests that 5-HT_2A_ and 5-HT_2C_ receptors may be directly involved in alcohol drinking due to their interaction with the mesolimbic dopaminergic system. The aim of the present study was to investigate the effects of 5-HT_2A_ and 5-HT_2C_ antagonists, alone or in combination, on the acquisition and expression (i.e., return to alcohol drinking after a period of abstinence/treatment) of voluntary alcohol drinking in male mice.

**Methods:**

Animals had intermittent access to alcohol (10% v/v) in a two-bottle choice procedure for 30 days (acquisition), and were then submitted to alcohol re-exposure sessions after periods of abstinence. Vehicle, the 5-HT_2A_ receptor antagonist M100907 (M100, 1 mg/kg) and/or the 5-HT_2C_ receptor antagonist SB242084 (SB, 1 mg/kg) were administered either prior to acquisition (Experiment 1) or during the abstinence period preceding re-exposure sessions (Experiment 2). During re-exposure tests, animals were submitted to the same conditions as during acquisition, with no treatments prior to those sessions.

**Results:**

Our findings show that combined treatment with 5-HT_2A_ and 5-HT_2C_ antagonists, but not treatment with the antagonists separately, reduced alcohol drinking and preference when administered immediately before acquisition (Experiment 1). Combined treatment with 5-HT_2A_ and 5-HT_2C_ antagonists after the establishment of voluntary alcohol drinking did not alter the expression of drinking behavior (Experiment 2). On the other hand, while post-acquisition treatment with a 5-HT_2A_ antagonist alone decreased alcohol intake and preference during re-exposure, co-administration of a 5-HT_2C_ antagonist blocked these effects.

**Discussion:**

Our findings suggest that 5-HT_2A_ and 5-HT_2C_ receptors differentially modulate the acquisition and expression of voluntary alcohol drinking in mice.

## 1 Introduction

Alcohol use disorder (AUD) continues to be a public health concern worldwide (The Lancet Gastroenterology Hepatology, 2024), with treatment options being only partially effective and relapse rates remaining high (Kurihara et al., 2023). While the field of AUD has made significant progress in understanding the neurobiological mechanisms underlying alcohol use (Koob and Volkow, 2010), the lack of broadly effective medications for AUD emphasizes the need for further studies investigating novel mechanisms that mediate alcohol use.

Accumulating evidence suggests that serotonin (5-HT) receptors, particularly the 5-HT_2A_ and 5-HT_2C_ subtypes, play key roles in modulating alcohol use and AUD (Marcinkiewcz, 2015). 5-HT_2A_ receptors are highly expressed in glutamatergic and dopaminergic neurons within the mesolimbic system (Howell and Cunningham, 2015). Activation of 5-HT_2A_ receptors can increase dopamine release in the nucleus accumbens and, consequently, enhance the abuse-related effects of alcohol (Alex and Pehek, 2007; Müller and Homberg, 2015). On the other hand, studies also show that 5-HT_2A_ receptor agonists can decrease alcohol drinking in rodents (Berquist and Fantegrossi, 2021; De Maurel et al., 1999), an effect that also has been reported after treatment with 5-HT_2A_ receptor antagonists (Ding et al., 2009). 5-HT_2C_ receptors, in contrast, generally exert an inhibitory effect on dopamine release due to their localization in GABAergic interneurons that mediate dopaminergic activity in the mesolimbic system (Howell and Cunningham, 2015). Accordingly, activation of 5-HT_2C_ receptors can reduce alcohol drinking by decreasing the activity of dopaminergic signaling (Di Matteo et al., 2008; Cunningham and Anastasio, 2014). However, intra-accumbal administration of a 5-HT_2C_ receptor antagonist also suppressed voluntary alcohol-drinking behavior in mice (Yoshimoto et al., 2012).

Together, these findings suggest that the specific roles of 5-HT_2A_ and 5-HT_2C_ receptors in alcohol drinking remain unclear. While studies have investigated the role of serotonin 5-HT_2A_ and 5-HT_2C_ receptors in the behavioral effects of alcohol (Marcinkiewcz, 2015), several findings show contradictory results and the majority of these studies investigated the effects of 5-HT_2A_ and 5-HT_2C_ receptor agonists (Castle and Flanigan, 2024), emphasizing the need for further studies elucidating the serotonergic mechanisms underlying alcohol drinking, particularly with antagonist approaches. In the present study, we sought to investigate not only the role of these receptors on voluntary alcohol drinking separately, but also whether interactions exist between these serotonergic mechanisms. By investigating their effects on both the acquisition and expression (i.e., return to alcohol drinking after a period of abstinence/treatment) of alcohol drinking, our goal was to identify whether specific 5-HT_2A_ and 5-HT_2C_ receptor mechanisms are involved in one or more phases of the alcohol drinking cycle. Understanding the interplay between these receptors could help inform the neurobiological mechanisms underlying alcohol use and the development of serotonergic pharmacotherapies for AUD.

## 2 Materials and methods

### 2.1 Animals

Three-month-old Swiss male mice from our own colony were used. Animals weighing 35–40 g were group-housed (10 per cage) for most of the study, except during self-administration sessions, during which animals were single-housed for 15 h every other day. When in groups, animals were housed in polypropylene cages (32 × 42 × 18 cm) under controlled temperature (22–23°C) and light/dark cycle (12 h light, 12 h dark; lights on at 06 h 40). Food and water were available *ad libitum* throughout the experiments. Animals were maintained according to the National Institutes of Health Guide for the Care and Use of Laboratory Animals (8th Edition, revised 2011) and in accordance with the Brazilian Law for Procedures for Animal Scientific Use (#11794/2008). The Institutional Animal Care and Use Committee of UESC approved the experimental procedures.

### 2.2 Drugs

The 5-HT_2A_ receptor antagonist M100907 (M100) and the 5-HT_2C_ receptor antagonist SB242084 (SB) were synthesized at the Drug Design and Synthesis Section, National Institute on Drug Abuse and National Institute on Alcohol Abuse and Alcoholism at the National Institutes of Health (Bethesda, MD, United States). M100 and SB (both 1 mg/kg, 0.1 mg/mL) were dissolved in sterile saline (0.9%) and administered in the form of a suspension due to solubility constraints, as noted in previous publications (Ansah et al., 2011; Knapp et al., 2004). M100, SB and their vehicle (Veh, saline) were administered intraperitoneally (i.p.). Alcohol 95% (Merck^®^) was diluted in water at a 10% water/volume drinking solution. The doses of M100 and SB were chosen based on studies showing significant reductions in alcohol-related behaviors at the dose of 1 mg/kg (Serra et al., 2022; Ko and Hwa, 2023).

### 2.3 Two-bottle choice alcohol drinking

Mice were given the opportunity to drink 10% alcohol using an intermittent access, two-bottle choice procedure. This protocol is based on previous studies showing that chronic intermittent access to alcohol is critical for escalation of drinking using a two-bottle choice procedure (Griffin III et al., 2009; Carrara-Nascimento et al., 2017). These protocols also include forced abstinence/treatment periods followed by re-exposure to alcohol drinking to mimic the chronic episodic nature of alcohol use in humans (Carrara-Nascimento et al., 2017). This protocol has been in place in our laboratory for several years for studying voluntary drinking of alcohol (Serra et al., 2022) and other drugs (Jovita-Farias et al., 2023; Kisaki et al., 2024).

The protocol was divided into 3 phases: acquisition, abstinence/treatment and re-exposure. For all phases, when animals were not in the individual drinking cages, animals remained group-housed in their home cages. Food was available *ad libitum* during all sessions. Experimental protocols are described in the next section.

For all experiments, consumption from each bottle (water or alcohol) was measured at the end of each session. Bottles were refilled with fresh water or fresh alcohol solutions before each session. Bottle sides were switched at every session. Each bottle of water or alcohol was weighed on a precision scale before and after the session to determine the consumption of water/alcohol. Animals were also weighed daily. To ensure data were not affected by liquid loss due to bottle leaks or evaporation, two bottles were left in an empty cage for 1 week, during which time liquid loss was measured and found to be less than 0.1 mL/day. After obtaining the final weight of each alcohol bottle, alcohol consumption in g/kg was calculated for each session using the following formula:

$\begin{array}{l} \text{Alcohol consumption in}\;g/\mathrm{kg}=[\text{Amount of alcohol}\;\\ \text{consumed}\;(g)\times \text{alcohol concentration of solution}\times \text{alcohol}\;\\ \text{density}]\backslash [\text{animal weight}\;(g)/1,000]. \end{array}$

To calculate each animal’s preference for the alcohol bottle compared to the water bottle, the following formula was used:

$\begin{array}{l} \text{Preference}=[\text{Amount of alcohol consumed}\;(g)/\\ \text{total consumption}\;(\text{water}+\text{alcohol solution})\;(g)]\times 100. \end{array}$

### 2.4 Experimental design

#### 2.4.1 Experiment 1: effects of 5-HT_2A_ and 5-HT_2C_ receptor antagonists on the acquisition of alcohol drinking

**Figure 1:**
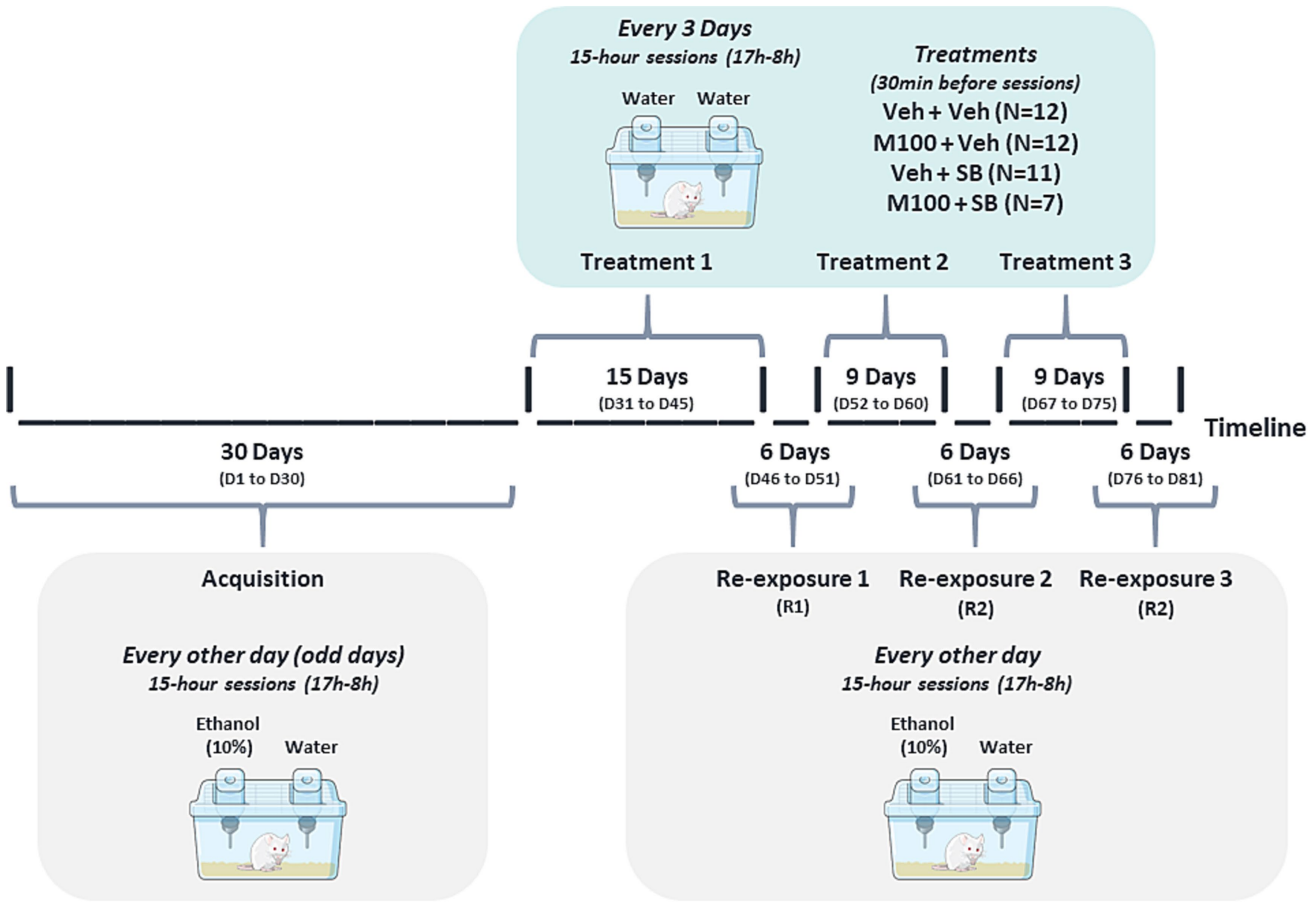
Experimental design for Experiment 1. Animals were submitted to the alcohol two-bottle choice procedure acquisition phase, during which they were exposed to individual cages with access to two bottles, one of water and one of 10% alcohol solution, for 15 h, every other day. During acquisition, animals were treated with vehicle (Veh, i.p.), the 5-HT_2A_ antagonist M100907 (M100, 1 mg/kg, i.p.) and/or the 5-HT_2C_ antagonist SB242084 (SB, 1 mg/kg, i.p.). After acquisition, animals were submitted to two abstinence phases, each followed by a re-exposure phase, during which animals were again exposed to alcohol.

Figure 1 illustrates the experimental design for Experiment 1. Every other day (odd days) for 30 days, 41 animals were housed individually in polypropylene boxes (30 × 19 × 13 cm) for 15 h (17 h00-08 h00) and given access to two bottles, one containing 10 mL of water and the other containing 10 mL of 10% alcohol solution. Thirty minutes before being placed in the individual boxes, animals were treated with Veh + Veh (*N* = 11), M100 + Veh (*N* = 9), Veh + SB (*N* = 10) or M100 + SB (*N* = 11).

Animals were then submitted to 2 abstinence periods, each followed by an alcohol re-exposure phase (R1 and R2). During abstinence days, animals were left undisturbed in their home-cages. The first abstinence period lasted 14 days and the second lasted 7 days. At the end of each abstinence period, animals were submitted to an alcohol re-exposure phase, being individually placed in the two-bottle choice cage with access to one bottle of water and one bottle of 10% alcohol solution, similarly to the acquisition phase. Each re-exposure phase lasted 6 days, with animals being re-exposed to alcohol every other day (total of 3 re-exposure sessions).

#### 2.4.2 Experiment 2: effects of 5-HT_2A_ and 5-HT_2C_ receptor antagonists on the expression of alcohol drinking

**Figure 2:**
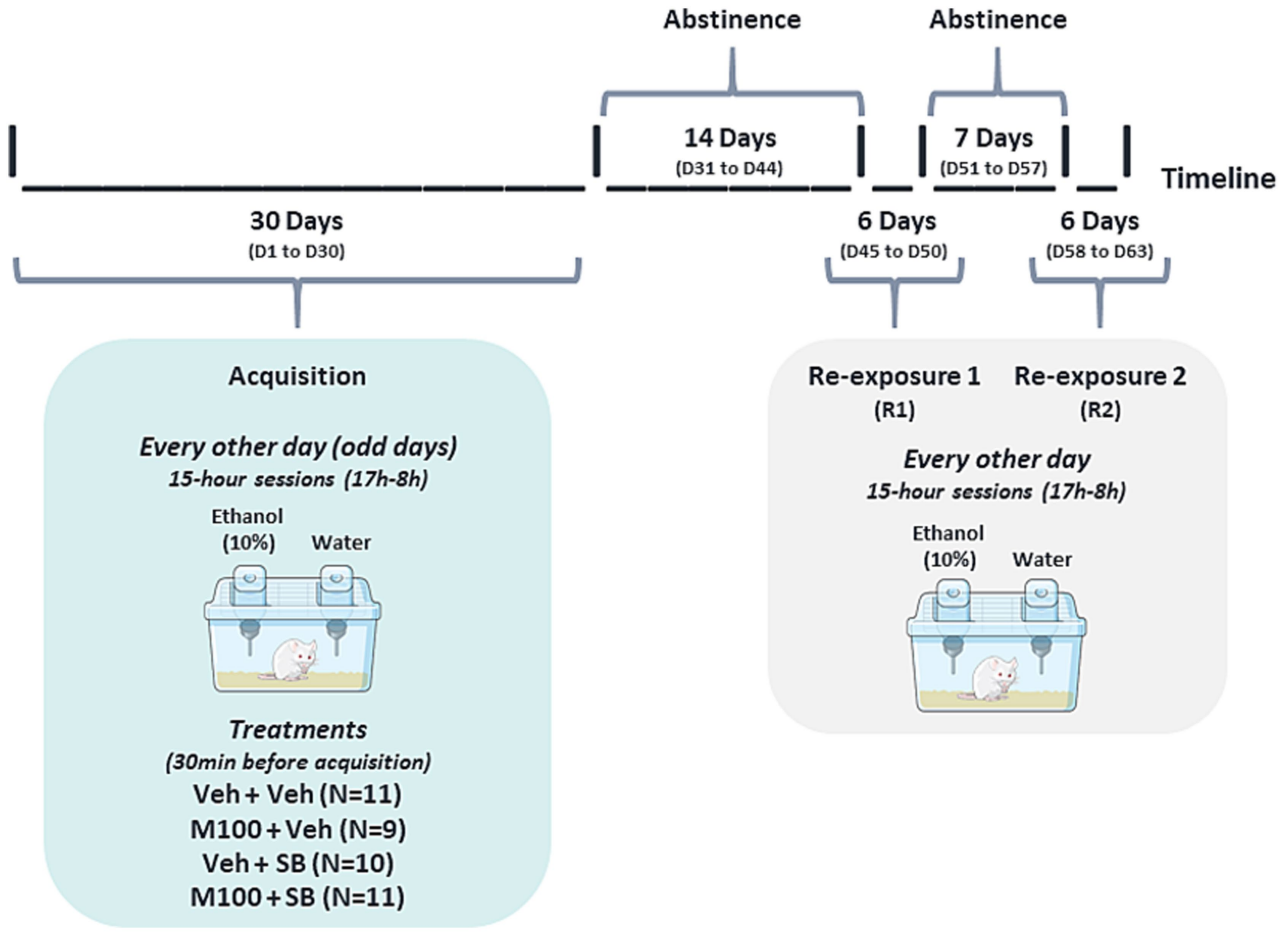
Experimental design for Experiment 2. Animals were submitted to the alcohol two-bottle choice procedure acquisition phase, during which they were exposed to individual cages with access to two bottles, one of water and one of 10% alcohol solution, for 15 h, every other day. At the end of the acquisition phase, animals were randomly assigned to 4 groups, being treated with vehicle (Veh, i.p.), the 5-HT_2A_ antagonist M100907 (M100, 1 mg/kg, i.p.) and/or the 5-HT_2C_ antagonist SB242084 (SB, 1 mg/kg, i.p.) during the treatment phases. Each treatment phase was followed by a re-exposure phase, during which animals were again exposed to alcohol.

Figure 2 Illustrates the experimental design for experiment 2. Every other day (odd days) for 15 days, 60 animals were housed individually in polypropylene boxes (30 × 19 × 13 cm) for 15 h (17 h00–08 h00) and given access to two bottles, one containing 10 mL of water and the other containing 10 mL of 10% alcohol solution. At the end of 15 days, the average alcohol intake between all animals was 1.57 g/kg, and 18 animals that did not reach 1.5 g/kg of alcohol intake were then excluded from the study. The remaining (42) animals with alcohol intake ≥ 1.5 g/kg were maintained in the study and continued to be submitted to the acquisition protocol for another 15 days (total of 15 acquisition sessions over the course of 30 days, with sessions happening every other day). The 42 animals were distributed evenly and randomly across experimental groups, with the exception of the co-administration group (M100 + SB), in which only 7 animals were included due to constraints related to drug availability.

Animals were then submitted to 3 treatment periods, each followed by 3 alcohol re-exposure sessions. During the first treatment period, animals were treated every 3 days with Veh + Veh (*N* = 12), M100 + Veh (*N* = 12), Veh + SB (*N* = 11) or M100 + SB (*N* = 7) for a period of 15 days (total of 5 treatment sessions). Thirty minutes after treatments, animals were individually placed in the two-bottle choice cage with access to 2 bottles of water. Forty-eight hours after the last treatment session, animals were submitted to the first alcohol re-exposure session (R1), being individually placed in the self-administration cage with access to one bottle of water and one bottle of 10% alcohol solution, similarly to the acquisition phase. Re-exposure sessions happened every other day for 6 days (total of 3 re-exposure sessions) before the next treatment phase. The 2 subsequent treatment phases followed the same protocol described for the first treatment phase, except they were shorter: 9 days each, with treatments every 3 days (3 treatments total per treatment phase). Each treatment phase was also followed by a 6-day re-exposure phase (R2 and R3), with re-exposure tests taking place every other day (3 re-exposures each).

### 2.5 Statistical analysis

Power analyses were conducted using Cohen’s d-statistic and GPower software based on our published findings using a similar experimental design (Serra et al., 2022; Kisaki et al., 2024). Based on these estimates, for a mixed factor ANOVA with drug treatment as the between-subjects factor, an effect size (f) of 0.50 is expected with sample sizes of *N* = 7 ([1-β] = 0.95, nonsphericity correction [ε] = 1.0). When possible, we have increased the sample size in case of attrition. All variables were checked for normality (Shapiro–Wilk test) and homogeneity of variances (Levene’s test). Data for the acquisition phase were grouped by 6-day periods (3 sessions/period), and data for the re-exposure test were also grouped as the 3 re-exposure sessions for each re-exposure phase. Statistical analyses were performed using analysis of variance (ANOVA), with or without repeated measures (RM), and sphericity was assumed for all RM tests. Within- and between-subject factors are defined for each analysis in the results section. Multiple comparisons were performed using Bonferroni *post hoc* tests. Analyses and graphic representations were performed using GraphPad Prism (v. 10.4.2). A probability of *p* < 0.05 was considered a statistically significant difference.

## 3 Results

### 3.1 Experiment 1: effects of 5-HT_2A_ and 5-HT_2C_ receptor antagonists on the acquisition of alcohol drinking

**Figure 3:**
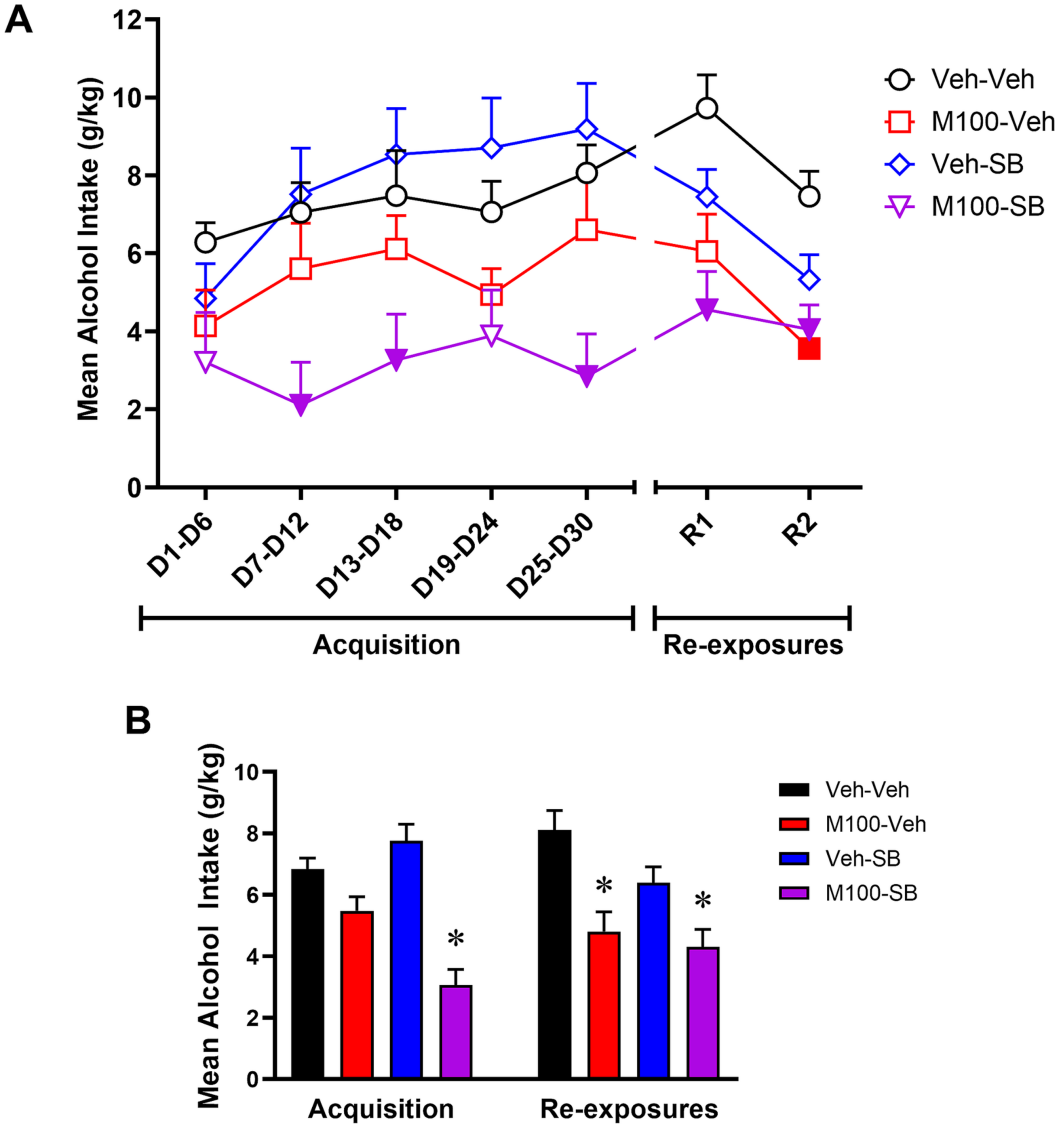
Mean alcohol intake (g/kg) during the acquisition of two-bottle choice alcohol self-administration and during alcohol re-exposures. Animals were treated before acquisition sessions with vehicle (Veh-Veh, *N* = 11), the 5-HT_2A_ antagonist M100907 (M100-Veh, 1 mg/kg, i.p., *N* = 9), the 5-HT_2C_ antagonist SB242084 (Veh-SB, 1 mg/kg, i.p., *N* = 10), or with a combination of M100907 and SB242084 (M100-SB, both 1 mg/kg, i.p., *N* = 11). **(A)** Alcohol intake over time, in which each time-point is an average of 3 sessions within the same phase (i.e., 3 acquisition sessions or 3 re-exposure sessions) during the 30-day intermittent access acquisition phase or the 2 re-exposure phases (R1 and R2, each preceded by an abstinence period); filled symbols represent *p* < 0.05 vs. control (Veh-Veh). **(B)** Mean alcohol intake (g/kg) across all days of each phase of the procedure (acquisition vs. re-exposure); *p < 0.05 vs. control (Veh-Veh). Data are shown as mean ± SEM.

A two-way ANOVA of the total alcohol intake in g/kg across the experimental phases (Figure 3A) with time (all time-points) as within-subject factor and treatment as between-subject factors showed a significant interaction between time and treatment [*F*(18, 222) = 2.20; *p* < 0.01]. Bonferroni’s *post hoc* analysis showed that animals treated with the combination of M100 + SB drank less alcohol compared to the control (Veh-Veh) group during D7–D12, D13–D18, D25–D30 and during both re-exposure tests (R1 and R2) (*p* < 0.05 s). While neither drug alone decreased alcohol drinking during acquisition, treatment with M100 significantly decreased alcohol drinking compared to control during the second re-exposure test (*p* < 0.05). A two-way ANOVA of total water intake across the experimental phases with time (all time-points) as within-subject factor and treatment as between-subject factors also showed a significant treatment effect [*F* (6, 228) = 2.39; *p* < 0.05], with *post hoc* tests showing that water intake was significantly increased in animals treated with the combination of M100 + SB compared to the control (Veh-Veh) group during the same time-points when alcohol intake was decreased (*p* < 0.05 s, data not shown), suggesting a shift in overall consumption from alcohol to water. These findings were also reflected in our analysis averaging alcohol intake (g/kg) across all days of each phase of the procedure (acquisition vs. re-exposure, Figure 3B). A two-way ANOVA with M100 treatment and SB treatment as between-subject factors showed a significant interaction between treatments during acquisition [*F*(1, 206) = 12.62; *p* < 0.001], with Bonferroni post hoc tests showing that treatment with the combination of M100 + SB significantly decreased alcohol intake during acquisition compared to control (*p* < 0.05). A similar two-way ANOVA for the re-exposure phase showed only a significant effect of M100 treatment [*F*(1, 80) = 20.47; *p* < 0.0001], but no effect of SB alone [*F*(1, 80) = 3.49; *p* = 0.06] or interaction between treatments [*F*(1, 80) = 1.05; *p* = 0.30]. Both the group treated with M100 alone and the group treated with the combination of M100 + SB showed decreased alcohol drinking during re-exposure compared to control (Bonferroni’s test, *p* < 0.05).

**Figure 4:**
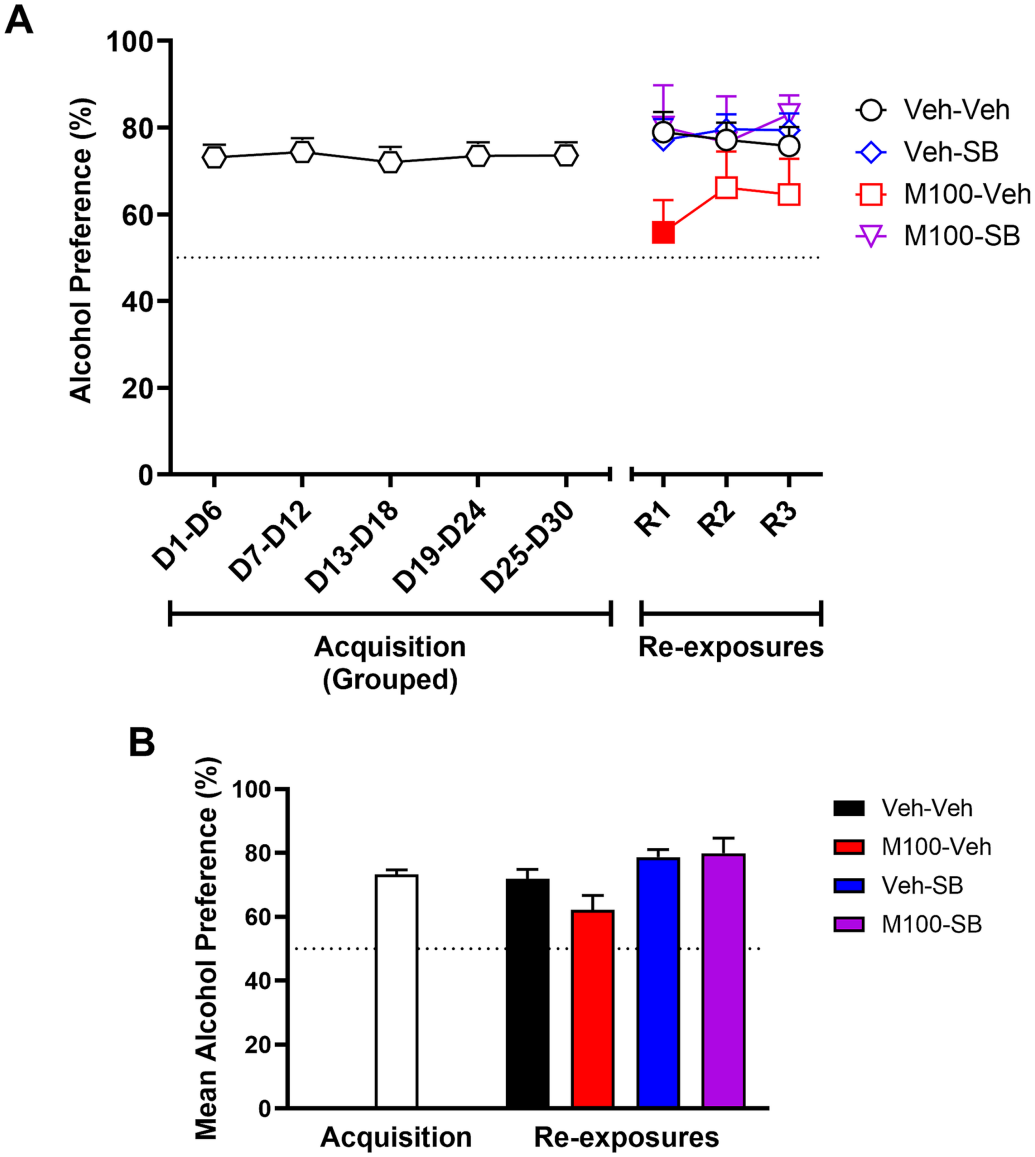
Preference (%) for the alcohol bottle during the acquisition of two-bottle choice alcohol self-administration and during alcohol re-exposures. Animals were treated before acquisition sessions with vehicle (Veh-Veh, *N* = 11), the 5-HT_2A_ antagonist M100907 (M100-Veh, 1 mg/kg, i.p., *N* = 9), the 5-HT_2C_ antagonist SB242084 (Veh-SB, 1 mg/kg, i.p., *N* = 10), or with a combination of M100907 and SB242084 (M100-SB, both 1 mg/kg, i.p., *N* = 11). **(A)** Alcohol preference over time, in which each time-point is an average of 3 sessions within the same phase (i.e., 3 acquisition sessions or 3 re-exposure sessions) during the 30-day intermittent access acquisition phase or the 2 re-exposure phases (R1 and R2, each preceded by an abstinence period); filled symbols represent *p* < 0.05 vs. control (Veh-Veh). **(B)** Mean alcohol preference across all days of each phase of the procedure (acquisition vs. re-exposure); Data are shown as mean ± SEM.

A two-way ANOVA of the preference for the alcohol bottle (%) across the experimental phases (Figure 4A) with time (all time-points) as within-subject factor and treatment as between-subject factors showed a significant effect of treatment [*F*(3, 37) = 8.00; p < 0.001], but no effect of time [*F*(6, 222) = 1.91; *p* = 0.07] or interaction time and treatment [*F*(18, 222) = 1.027; *p* = 0.43]. The control group showed a preference (greater than 50%) for the alcohol bottle during both the acquisition and the re-exposure phases. Bonferroni’s *post hoc* analysis showed that animals treated with the combination of M100 + SB showed decreased alcohol preference compared to controls at all time-points during acquisition and re-exposure (*p* < 0.05 s). In fact, animals treated with the drug combination did not show alcohol preference (>50%) at any time-point in the study. Similar results were observed in our analysis averaging alcohol preference (%) across all days of each phase of the procedure (acquisition vs. re-exposure, Figure 4B). A two-way ANOVA with M100 treatment and SB treatment as between-subject factors showed a significant interaction between treatments during acquisition [*F*(1, 200) = 4.52; *p* < 0.05], with Bonferroni post hoc tests showing that treatment with the combination of M100 + SB significantly decreased alcohol intake during acquisition compared to control (*p* < 0.05). A similar two-way ANOVA for the re-exposure phase showed a significant effect of M100 treatment [*F*(1, 80) = 12.08; *p* < 0.001] and of SB treatment [*F*(1, 80) = 8.47; *p* < 0.01], but no interaction between treatments [*F*(1, 80) = 0.01; *p* = 0.94]. Only the group treated with the combination of M100 + SB showed significantly lower alcohol preference during re-exposure compared to control (Bonferroni’s test, *p* < 0.05).

### 3.2 Experiment 2: effects of 5-HT_2A_ and 5-HT_2C_ receptor antagonists on the expression of alcohol drinking

**Figure 5:**
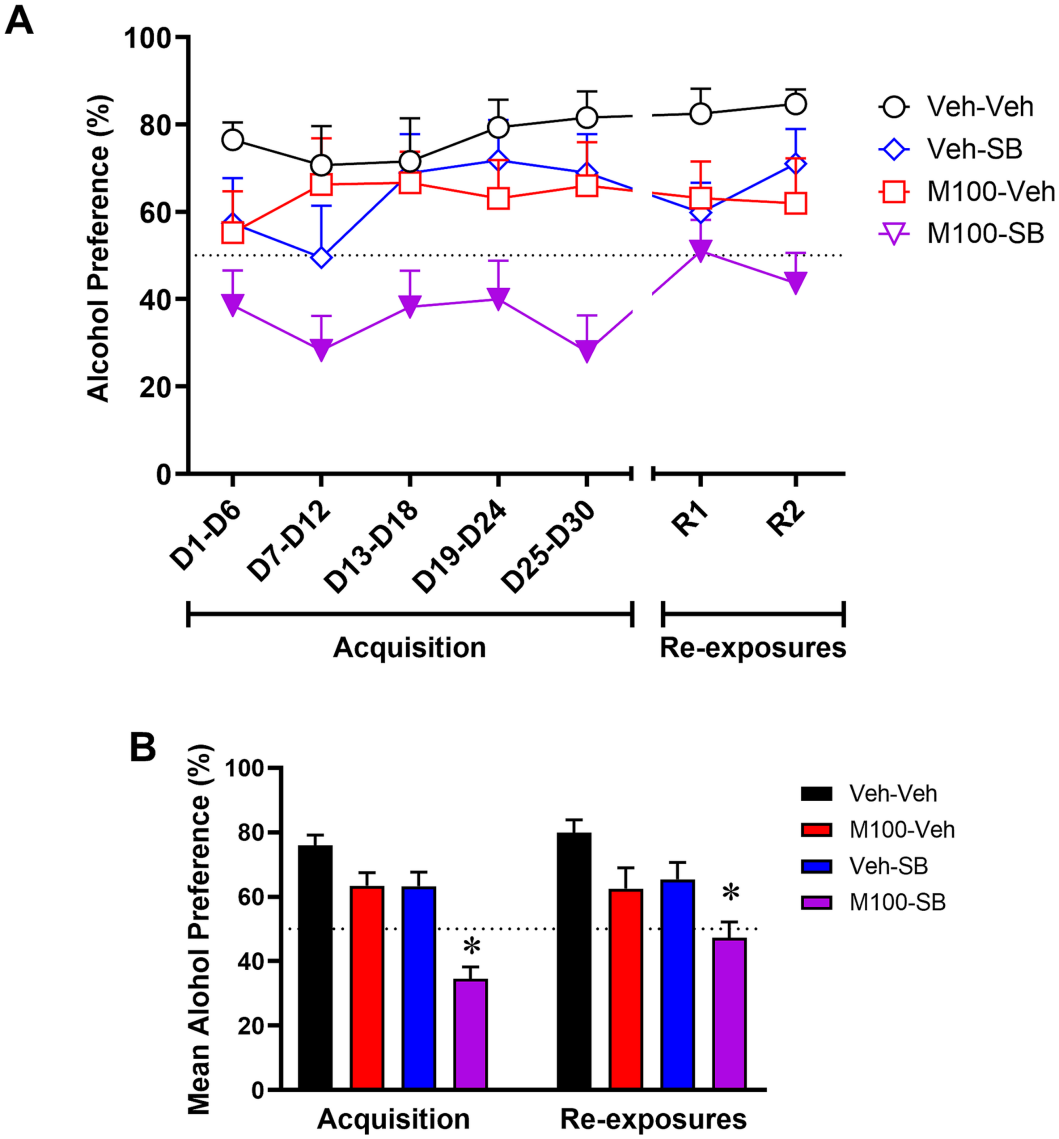
Mean alcohol intake (g/kg) during the acquisition of two-bottle choice alcohol self-administration and during alcohol re-exposures. Because all groups were submitted to the same experimental conditions during acquisition, acquisition phase data were grouped for graphical representation. Animals were then treated during intervals before each re-exposure phase (R1, R2, and R3) with vehicle (Veh-Veh, *N* = 12), the 5-HT_2A_ antagonist M100907 (M100-Veh, 1 mg/kg, i.p., *N* = 12), the 5-HT_2C_ antagonist SB242084 (Veh-SB, 1 mg/kg, i.p., *N* = 11), or with a combination of M100907 and SB242084 (M100-SB, both 1 mg/kg, i.p., *N* = 7). **(A)** Alcohol intake over time, in which each time-point is an average of 3 sessions within the same phase (i.e., 3 acquisition sessions or 3 re-exposure sessions) during the 30-day intermittent access acquisition phase or the 3 re-exposure phases (R1, R2, and R3, each preceded by an abstinence period); filled symbols represent *p* < 0.05 vs. control (Veh-Veh). **(B)** Mean alcohol intake (g/kg) across all days of each phase of the procedure (acquisition vs. re-exposure); *p < 0.05 vs. control (Veh-Veh). Data are shown as mean ± SEM.

Because all groups were submitted to the same experimental conditions during the acquisition phase of Experiment 2, we grouped the acquisition phase data for graphical representation. To confirm lack of group differences before beginning of treatments, a two-way ANOVA of the acquisition phase with time (all time-points) as within-subject factor and group as between-subject factors showed no significant effects of time [*F*(4, 172) = 0.18; *p* = 0.94], group [*F*(3, 43) = 1.6; *p* = 0.18] or time vs. group interactions [*F*(12, 172) = 1.11; *p* = 0.34] for alcohol intake (g/kg). A two-way ANOVA of the total alcohol intake in g/kg across the re-exposure tests (Figure 5A) with time (R1, R2, and R3) as within-subject factor and treatment as between-subject factors showed a significant effect of treatment [*F*(3, 42) = 2.70; *p* < 0.05], but no effect of time [*F*(2, 84) = 0.28; *p* = 0.75] or interaction between the factors [*F* (6, 84) = 0.39; *p* = 0.87]. Bonferroni’s *post hoc* analysis showed that animals treated with M100 alone showed decreased alcohol intake during all three re-exposure phases compared to the control (Veh-Veh) group (*p* < 0.05 s). SB alone or a combination treatment with M100 + SB had no significant effects on alcohol drinking (*p* > 0.05). A two-way ANOVA of the total water intake across the re-exposure tests with time (R1, R2, and R3) as within-subject factor and treatment as between-subject factors showed a significant effect of treatment [*F*(3, 42) = 3.42; *p* < 0.05], with *post hoc* tests showing that water intake was significantly increased in animals treated with M100 alone compared to the control (Veh-Veh) group during the same time-points when alcohol intake was decreased (*p* < 0.05 s, data not shown), suggesting a shift in overall consumption from alcohol to water. Interestingly, in our analysis averaging alcohol intake (g/kg) across all days of each phase of re-exposure phase (Figure 5B), a two-way ANOVA with M100 treatment and SB treatment as between-subject factors showed a significant interaction between treatments during re-exposure [*F*(1, 135) = 5.44; *p* < 0.05]. Bonferroni *post hoc* tests showed that only treatment with M100 significantly decreased alcohol intake compared to control (*p* < 0.05).

**Figure 6:**
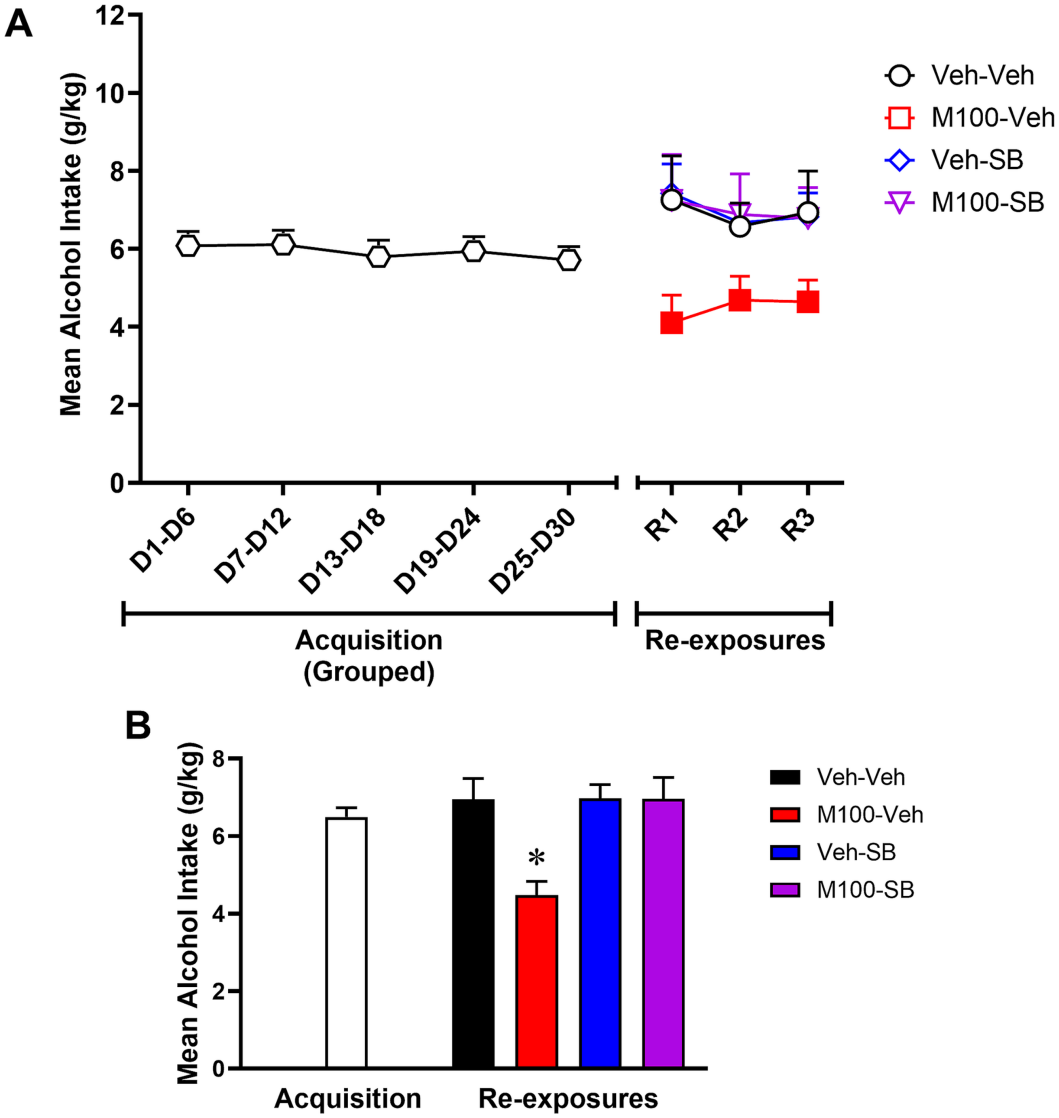
Preference (%) for the alcohol bottle during the acquisition of two-bottle choice alcohol self-administration and during alcohol re-exposures. Because all groups were submitted to the same experimental conditions during acquisition, acquisition phase data were grouped for graphical representation. Animals were then treated during intervals before each re-exposure phase phase (R1, R2, and R3) with vehicle (Veh-Veh, *N* = 12), the 5-HT_2A_ antagonist M100907 (M100-Veh, 1 mg/kg, i.p., *N* = 12), the 5-HT_2C_ antagonist SB242084 (Veh-SB, 1 mg/kg, i.p., *N* = 11), or with a combination of M100907 and SB242084 (M100-SB, both 1 mg/kg, i.p., *N* = 7). **(A)** Alcohol preference over time, in which each time-point is an average of 3 sessions within the same phase (i.e., 3 acquisition sessions or 3 re-exposure sessions) during the 30-day intermittent access acquisition phase or the 3 re-exposure phases (R1, R2, and R3, each preceded by an abstinence period); filled symbols represent *p* < 0.05 vs. control (Veh-Veh). **(B)** Mean alcohol preference across all days of each phase of the procedure (acquisition vs. re-exposure); *p < 0.05 vs. control (Veh-Veh). Data are shown as mean ± SEM.

All groups showed a preference (>50%) for the alcohol bottle at all stages of the acquisition phase. To confirm lack of group differences before beginning of treatments, a two-way ANOVA of the acquisition phase with time (all time-points) as within-subject factor and group as between-subject factors showed no significant effects of time [*F*(4, 172) = 0.06; *p* = 0.99], group [*F*(3, 43) = 4.37; *p* = 0.09] or time vs. group interactions [*F*(12, 172) = 1.22; *p* = 0.27] for alcohol preference (%). A two-way ANOVA of the alcohol preference across the re-exposure tests (Figure 6A) with time (R1, R2, and R3) as within-subject factor and treatment as between-subject factors showed a significant effect of treatment [*F*(3, 41) = 2.53; *p* < 0.05], but no effect of time [*F*(2, 82) = 0.43; *p* = 0.64] or interaction between the factors [*F*(6, 82) = 0.82; *p* = 0.55]. Bonferroni’s post hoc analysis showed that animals treated with M100 alone showed decreased alcohol preference during re-exposure test R1 compared to the control group (*p* < 0.05). No significant effects were seen for the other re-exposure tests, and SB alone or a combination treatment with M100 + SB had no significant effects on alcohol preference (*p* > 0.05). In our analysis averaging alcohol preference (%) across all days of the re-exposure phase (Figure 6B), a two-way ANOVA with M100 treatment and SB treatment as between-subject factors showed no significant effects of either treatment (M100: [*F*(1, 122) = 0.72; *p* = 0.39]; SB: [*F*(1, 122) = 8.31; *p* = 0.060]) or interaction between treatments [*F*(1, 122) = 1.33; *p* = 0.25].

## 4 Discussion

Our findings show that neither 5-HT_2A_ nor 5-HT_2C_ antagonism was sufficient to reduce alcohol drinking when administered immediately before the acquisition phase of the two-bottle choice procedure. However, combined treatment with the two serotonin receptor antagonists significantly decreased alcohol intake and preference, suggesting an additive or synergistic interaction between the two receptors on voluntary alcohol drinking. These effects resulted in decreased alcohol intake and preference after treatment discontinuation (re-exposure phase). Interestingly, combined treatment with 5-HT_2A_ and 5-HT_2C_ antagonists after the establishment (acquisition) of voluntary alcohol drinking did not alter the expression (re-exposure) of drinking behavior. In fact, while post-acquisition treatment with a 5-HT_2A_ antagonist alone decreased alcohol intake and preference during re-exposure, co-administration of a 5-HT_2C_ antagonist blocked these effects.

Our findings showing that co-administration of a 5-HT_2C_ antagonist blocked the effects of a 5-HT_2A_ antagonist on alcohol-related behaviors are in agreement with the proposed oppositional control of 5-HT_2A_ and 5-HT_2C_ receptor ligands over the addiction-related effects of drugs (Howell and Cunningham, 2015). Because of the regional distribution of those receptors in the brain, they exert opposing effects on the dopaminergic mesolimbic system (Bortolozzi et al., 2005; Manvich et al., 2012a,b; Murnane et al., 2013; Berro et al., 2017; Valencia-Torres et al., 2017). The ventral tegmental area (VTA) consists of dopaminergic neurons that project to both the nucleus accumbens and the prefrontal cortex (PFC). The PFC consists of pyramidal glutamatergic neurons that project to the nucleus accumbens and the VTA. Both VTA and PFC neurons are locally regulated by GABAergic interneurons. Importantly, VTA dopaminergic neurons and PFC glutamatergic neurons predominantly express 5-HT_2A_ receptors, and blockade of those receptors would be expected to decrease VTA dopamine neurotransmission (Bortolozzi et al., 2005). On the other hand, VTA and PFC GABAergic interneurons predominantly express 5-HT_2C_ receptors. Blockade of 5-HT_2C_ receptors would be expected to decrease the activity of GABAergic interneurons, disinhibiting VTA and PFC excitatory neurons (Howell and Cunningham, 2015) and, ultimately, offsetting 5-HT_2A_ receptor antagonism-induced decreased mesolimbic dopamine activity. In agreement, our findings elucidate previous studies showing that nonselective 5-HT_2A_/_2C_ receptor antagonists, such as ritanserin, failed to alter alcohol preference after the establishment of alcohol drinking in rats (Myers and Lankford, 1993; Rammsayer and Vogel, 1994).

The contrasting effects observed with the combination of 5-HT_2A_ and 5-HT_2C_ antagonists before acquisition vs. re-exposure suggest that different serotonergic mechanisms may mediate initial vs. long-term alcohol drinking. This is further corroborated by the mixed results reported on alcohol drinking after treatment with nonselective 5-HT_2A_/_2C_ receptor antagonists. In general, 5-HT_2_ antagonists seem to significantly decrease alcohol intake when administered during early alcohol drinking (e.g., 7 days of alcohol drinking before antagonist treatment; Panocka and Massi, 1992), while having no effects when administered after more than 2 weeks of daily alcohol intake (Myers and Lankford, 1993; Myers et al., 1993). Importantly, studies also suggest that the effects of non-selective 5-HT_2C_ receptor antagonists on alcohol drinking are dependent on increased synaptic availability of 5-HT in the nucleus accumbens (Yoshimoto et al., 2012). Given the evidence showing that nucleus accumbens serotonin levels are increased after acute alcohol administration (Yoshimoto et al., 1992) yet decreased after chronic alcohol drinking (McBride et al., 1995; Thielen et al., 2004), it is plausible that changes in serotonin and serotonin receptor availability mediate the effects of 5-HT_2A_/_2C_ receptor antagonists on acute vs. chronic alcohol drinking, as seen in the present study. While studies have shown that chronic alcohol exposure leads to increased expression of 5-HT_2C_ receptors in the nucleus accumbens, much less is known regarding changes in 5-HT_2A_ receptor expression after chronic alcohol use, emphasizing the need for further studies to determine specific brain regions and cell types that are important for mediating the effects of 5-HT_2A_ and 5-HT_2C_ compounds on alcohol drinking (Castle and Flanigan, 2024).

Of note, while treatment with the 5-HT_2A_ antagonist alone did not alter acquisition of alcohol drinking, it decreased alcohol drinking during re-exposure both when it was administered before and after acquisition. Those findings further suggest that 5-HT_2A_-mediated serotonin neurotransmission is important for the expression of the alcohol drinking. In agreement, 5-HT_2A_ agonists have been shown to potentiate alcohol-induced excitation of dopamine neurons in the mesolimbic system (Brodie et al., 1995), while a 5-HT_2A_ receptor antagonist attenuated operant alcohol self-administration in rats (Ding et al., 2009).

Together, our findings suggest that that 5-HT_2A_ and 5-HT_2C_ receptors differentially modulate the acquisition and expression of voluntary alcohol drinking in mice. Specifically, administration of a 5-HT_2A_ receptor antagonist decreased the expression of alcohol drinking and preference regardless of the timing of this intervention. While the 5-HT_2C_ receptor antagonist did not have significant effects on its own, it did modulate the effects of the 5-HT_2A_ receptor antagonist, enhancing them when administered during early alcohol drinking (acquisition) and reversing them when administered after long-term alcohol intake (after acquisition). These findings further emphasize the importance of 5-HT_2_ receptor in modulating acute and chronic alcohol drinking, and also suggest that 5-HT_2A_ and 5-HT_2C_ receptor antagonists may be promising therapeutic agents for the treatment of alcohol use disorder. The clinical translation of 5-HT_2_ receptor antagonists for the treatment of alcohol use disorder has been limited to date (for review, see Castle and Flanigan, 2024). Importantly, studies have shown that the nonselective 5-HT_2A/2C_ receptor antagonist ritanserin did not alter mood, sleep quality, vigilance states or social functioning in individuals with alcohol use disorder (Wiesbeck et al., 2000), suggesting limited side effects of combination serotonergic strategies in this population. However, the efficacy of serotonergic compounds for the treatment of alcohol use disorder warrants further clinical investigation. Of note, an important limitation of the present study is the use of male mice only. Future studies are needed to investigate whether similar effects would be observed in females, particularly considering that sex differences have been reported for both alcohol drinking and brain serotonin systems, although evidence for sex differences in 5-HT_2A_ and 5-HT_2C_ receptor expression is scarce (Castle and Flanigan, 2024).

## Data Availability

The raw data supporting the conclusions of this article will be made available by the authors, without undue reservation.
